# The effect of glucagon-like peptide-1 and glucose dependent insulinotropic polypeptide receptor agonists on neurogenesis, differentiation, and plasticity (Neuro-GDP): potential mechanistically informed therapeutics in the treatment and prevention of mental disorders

**DOI:** 10.1017/S1092852925000124

**Published:** 2025-02-18

**Authors:** Roger S. McIntyre, Natalie Rasgon, Joseph Goldberg, Sabrina Wong, Gia Han Le, Rodrigo B. Mansur, Joshua D. Rosenblat, Kayla M. Teopiz, Stephen M. Stahl

**Affiliations:** 1 Department of Psychiatry, University of Toronto, Toronto, Canada; 2 Department of Psychiatry and Behavioral Sciences, Stanford School of Medicine, Rockefeller University; 3 Icahn School of Medicine at Mount Sinai, New York, NY; 4 Brain and Cognition Discovery Foundation, Toronto, Canada; 5 Department of Pharmacology & Toxicology, University of Toronto, Toronto, Canada; 6 Institute of Medical Science, University of Toronto, Toronto, Canada; 7 Department of Psychiatry and Neuroscience, University of California Riverside

**Keywords:** Neuroplasticity, neuroprotection, apoptosis, glucagon-like peptide-1 (GLP-1), glucose-dependent insulinotropic polypeptide (GIP)

## Abstract

Glucagon-like peptide-1 (GLP-1) and glucose-dependent insulinotropic polypeptide (GIP) receptor agonists (RAs) mimic naturally occurring GLP-1 and GIP and are highly effective anti-diabetic and anti-obesity agents. In addition to their robust acute and long-term effects on weight, metabolism, and blood pressure, these agents also reduce cardiovascular mortality as well as stroke risk and associated consequences. A replicated and convergent body of preclinical evidence also indicates that incretin receptor agonists activate molecular effectors critical to neuroplasticity, neuroprotection, and anti-apoptosis. Herein, we propose that GLP-1 RAs and GIP RAs are promising transdiagnostic mechanistically informed therapeutics in the treatment and prevention of multiple domains of psychopathology, including general cognitive, reward, and motivation systems and mental disorders. Major neurocognitive disorders (eg, Alzheimer’s Disease, Parkinson’s Disease), alcohol and substance use disorders, traumatic brain injury, and depressive disorders are near-term therapeutic targets. In addition, GLP-1 RAs and GIP RAs have robust effects on comorbidities that differentially affect persons with mental disorders (eg, cardiovascular, cerebrovascular, and metabolic disorders) and psychotropic drug-related weight gain.

## Introduction

The mechanism of action of antidepressants is not fully ascertained. It is hypothesized that antidepressant agents alleviate symptoms in depressive disorders by triggering molecular cascades integral to neuroplasticity, neuroprotection, and anti-apoptosis (NNA).^1^ ^,^^2^ The aforementioned molecular and cellular effects collectively modulate synaptic connection and strength as well as resting-state functional connectivity (RSFC) in discrete neural circuits and networks subserving the phenomenology of depressive disorders (eg, default mode network).^3^ ^–^^6^

Glucagon-like peptide-1 (GLP-1 RA) and glucose-dependent insulinotropic polypeptide receptor agonists (GIP RA), herein referred to as incretin receptor agonists (IRAs), mimic naturally occurring GLP-1 and GIP and are highly effective antidiabetic and anti-obesity agents.^7^ In addition to their robust acute and long-term effects on weight, metabolism, and blood pressure, these agents also reduce cardiovascular mortality as well as stroke risk and associated consequences.^7^ ^–^^9^ During the past decade, a replicated and convergent body of preclinical evidence also indicates that IRAs activate molecular effectors critical to NNA.

Herein, we propose that IRAs are promising treatments for mental disorders that engage brain targets critical to NNA known to subserve transdiagnostic phenomenology, notably general cognitive, reward, and motivation systems. We succinctly synthesize extant evidence but do not intend a review of the topic as multiple comprehensive reviews have been previously published.^10^ ^–^^19^ Instead, we attempt to provide a mechanistic framework for considering IRAs as putative mechanistically informed therapeutics for disparate mental disorders. Articles selected for citation are articles that were determined by authors to be most impactful either as original research or as a synthesis of available research.

## GLP-1 and GIP physiology and pharmacology

Glucagon-like peptide-1 is the product of preproglucagon encoded in intestinal L-cells as well as in neurons of the nucleus tractus solitarius (NTS). GLP-1 receptors are G-protein-coupled receptors (GPCRs) and are expressed in many human tissues, including the central nervous system (CNS; eg, hippocampus, hypothalamus, and cortex).^20^ GLP-1 receptors are expressed on endothelial cells, neurons, astrocytes, and microglia.^21^ ^–^^23^

Neuronal projections from the NTS to the paraventricular nucleus (PVN) of the hypothalamus facilitate reductions in food intake and behavior.^24^ ^,^^25^ GLP-1-producing neurons in the NTS also project to mesolimbic nuclei [eg, ventral tegmental area (VTA) and nucleus accumbens (NAcc)] and cortical structures.^20^ The aforementioned provides the basis for targeting these systems in the treatment and prevention of mental disorders.^26^

GIP is secreted by enteroendocrine K-cells, whose cognate receptor is also a GPCR.^27^ ^,^^28^ Whether GIP is synthesized in the CNS remains uncertain. Mixed evidence suggests that mRNA for GIP may be detected in select brain regions (eg, hippocampus).^29^ Notwithstanding, GIP receptors are expressed across disparate brain regions (eg, cortex, hippocampus, striatum).^27^ ^,^^30^ GIP receptor gene knockout results in reduced hippocampal NNA and impairs learning and memory in murine models.^27^ ^,^^31^

## GLP-1 and GIP effects on neuro-genesis, -differentiation, - plasticity (Neuro-GDP) (Figure 1)(Figure 2)

Glucagon-like peptide-1 receptor agonists activate multiple signal transduction pathways relevant to neuro-genesis, -differentiation, -plasticity (Neuro-GDP).^32^ For example, endogenous GLP-1, GIP, and GLP-1 RAs (eg, liraglutide) increase synthesis of cAMP response element-binding protein (CREB), brain-derived neurotrophic factor (BDNF), glial-derived neurotrophic factor (GDNF), and nerve growth factor (NGF) via PI3K-Akt activation.^33^ ^–^^35^ Moreover, GLP-1 promotes neuronal progenitor cell differentiation and neurite outgrowth.^36^ ^,^^37^

**Figure 1.**
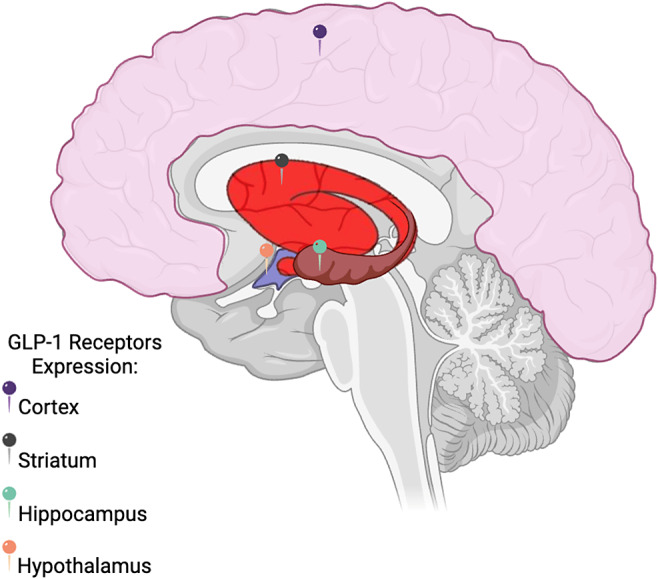
Distribution of GLP-1 receptors in the human central nervous system. Relevant structures that express GLP-1 receptors are highlighted.

**Figure 2.**
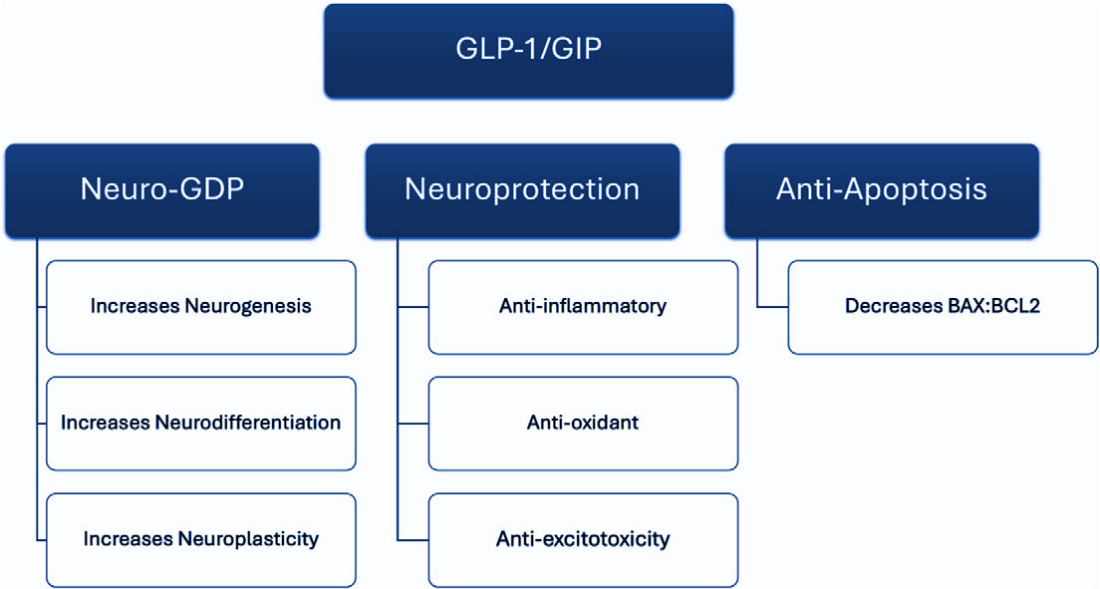
GLP-1 and GIP effects on neuro-genesis, -differentiation, - plasticity (GDP), neuroprotection and anti-apoptosis.

In rodent models and humans, GLP-1-mediated secretion of BDNF increases synaptic density in the hippocampus.^38^ ^–^^41^ It is hypothesized that trophic and plasticity effectors triggered by GLP-1 RAs mediate effects on cognition and/or motor function reported in Alzheimer’s and Parkinson’s Disease rodent models.^42^ ^–^^48^

Preclinical evidence indicates that liraglutide prevents reductions in phosphorylation levels of mTORC1 and BDNF expression in rat hippocampal structures exposed to neurotoxic levels of dexamethasone.^49^ The effect of liraglutide on BDNF expression and hippocampal dendrite length and spine density is blocked by rapamycin or the α-amino-3-hydroxy-5-methyl-4-isoxazolepropionic acid receptor (AMPA) receptor antagonist, 2,3-dioxo-6-nitro-1,2,3,4-tetrahydrobenzo[f]quinoxaline-7-sulfonamide (NBQX).^49^

Reduced long-term potentiation (LTP), increased long-term depression (LTD), and alterations in RSFC within and between brain circuits and networks are replicated findings in persons with depressive disorders.^50^ ^–^^52^ Glucagon-like peptide-1 receptor agonists rapidly increase excitatory postsynaptic currents and LTP.^53^ Glutamatergic availability and function are integral to LTP and synaptic strength.^54^

Glucagon-like peptide-1 receptor agonism modulates glutamatergic signaling by increasing AMPA trafficking, mTOR activation, and the transcription of synaptic proteins.^55^ ^–^^57^ In addition to the direct effects of GLP-1 RAs on molecular systems relevant to GDP, it is also observed that liraglutide increases intrinsic connectivity in bilateral hippocampal, medial-frontal, and lateral occipital regions, which inversely correlated with measures of insulin resistance in persons at genetic risk for Alzheimer’s Disease.^58^

Similar to the aforementioned effects of GLP-1 RAs on NNA, it is separately reported that GIP independently activates NNA systems. For example, GIP receptor knockout reduces neurogenesis and neurodifferentiation in the dentate gyrus of the hippocampus.^59^ In addition, GIP increases hippocampal synaptic number and plasticity effects in murine models.^60^ ^,^^61^

## GLP-1 and GIP neuroprotective effects: anti-inflammatory and antioxidant

It is further reported that GLP-1 RAs exert neuroprotective effects via modulating immune-inflammatory processes and redox balance.^43^ ^,^^62^ ^–^^66^ Pro-inflammatory processes and redox imbalance are implicated in the pathogenesis and progression of depressive disorders.^67^ Glucagon-like peptide-1 receptors are expressed on myeloid cells including monocytes, macrophages and glia (eg, microglia) where they modulate immune-inflammatory and redox balance systems.^68^ ^,^^69^ It is known that GLP-1 RAs decrease circulating C-reactive peptide (CRP), matrix metalloproteinase-9, monocyte chemoattractant protein-1, toll-like receptor 4 (TLR4), JNK-1 expression, nuclear factor-B as well as pro-inflammatory cytokines (eg, interleukin-6; IL-6) in human subjects.^70^

Glucagon-like peptide-1 is also synthesized and released in response to exposure to lipopolysaccharide.^71^ Glucagon-like peptide-1 receptor agonists also affect glial homeostasis and reactivity insofar as they induce less transcription of pro-inflammatory markers.^72^ ^,^^73^ Finally, it has been observed that GLP-1 RAs decrease blood-brain barrier (BBB) permeability under toxic conditions.^69^ ^,^^74^ ^,^^75^

The brain is especially susceptible to redox imbalance (ie, reactive oxygen species; ROS) due to its high oxygen utilization, lipid content, and relatively low endogenous antioxidant capacity.^67^ Oxidative stress compromises neuronal and glial integrity, viability, and function and is hypothesized to be integral to the pathoetiology of depressive and other mental disorders.^76^ ^,^^77^

Glucagon-like peptide-1 receptor agonists exert direct and indirect regulatory effects on redox imbalance.^78^ The antioxidant effects of GLP-1 RAs are observed whether oxidative stress is activated by glutamate or arachidonic acid.^78^ A replicated finding is that the antioxidant effects of GLP-1 RAs are mediated via their effect on ERK5/CREB signaling.^78^

Similar to GLP-1, disparate local and systemic anti-inflammatory effects are reported following GIP receptor activation. For example, GIP receptor activation results in decreased mRNA expression of macrophage chemoattractant protein 1 (MCP-1), vascular cell adhesion molecule 1 (VCAM-1), and intercellular adhesion molecule 1 (ICAM) as well as decreased circulating levels of interleukin IL-1β and TNFα.^79^ GIP knockout models report decrease in circulating monocytes and neutrophils, suggesting direct effects on hematopoietic lines.^80^ Neuroprotective effects of GIP are hypothesized to be mediated via their anti-inflammatory effects.^81^ The effect of GIP on redox balance is also replicated with observations of increases in glutathione peroxidase (GPX) and superoxide dismutase (SOD1) levels as well as decreased reactive oxygen species and release of nitric oxide.^82^

Beneficial effects of IRAs against post-stroke excitotoxicity as well as prevention involve many of the effectors previously covered.^9^ IRAs affect glutamatergic signaling via ionotropic receptor modulation. For example, it is separately reported that *N*-Methyl-D-Aspartate (NMDA) receptor antagonism increases GLP-1 synthesis and release improving glucose-insulin homeostasis in persons treated with NMDA antagonists (eg, dextromethorphan).^83^ ^–^^85^ Glucagon-like peptide-1 receptor agonists also modulate RSFC within and between circuits relevant to salience and cognitive control in persons with obesity and/or type 2 diabetes mellitus (T2DM).^86^

## Anti-apoptotic effects of GLP-1 and GIP

The pro-apoptotic BAX proteins are regulated by caspases and countered by the increased availability of SOD1, catalase (CAT), and GPX.^87^ Indirectly, GLP-1 receptor agonism modulates redox imbalance by reducing advanced glycation endproducts (AGE), malondialdehyde (MDA), and thiobarbituric acid reactive substances (TBARS).^87^ ^,^^88^. Glucagon-like peptide-1 receptor agonists, as well as dual GLP-1 agonists, reduce caspase-3 and BAX activity while simultaneously increasing Bcl-2 activity.^31^ ^,^^89^

## Clinical corollaries

Glucagon-like peptide-1 receptor agonists (eg, exenatide, liraglutide, semaglutide) and GLP-1/GIP co-agonists (eg, tirzepatide) are detectable in the brain and have differential CNS penetrance when using murine models.^90^ ^,^^91^ It remains uncertain the extent to which IRAs meaningfully penetrate (ie, target engagement) the BBB in human subjects and whether differential pharmacokinetics exist among the IRAs with respect to brain concentration.^92^ Preliminary evidence also suggests the benefit of IRAs in the treatment and/or prevention of alcohol-use, major neurocognitive (eg, Alzheimer’s Disease), Parkinson’s Disease, traumatic brain injury, and nicotine use.^12^ ^,^^26^ ^,^^93^ ^–^^111^ A critical issue bridging preclinical study results to critical translation is posology and route of administration. Doses implemented in many of the preclinical models approximate comparable human doses but are not identical. Moreover, the route of administration in animals is similar to (eg, parenteral) human administration of IRAs, although it is recognized that there is a growing interest in the oral administration of IRAs in humans. In addition, it is unknown whether the dosing of IRAs that are effective in the prevention and treatment of mental disorders is similar to anti-diabetic and anti-obesity dosing.

An emerging literature has also examined antidepressant efficacy associated with GLP-1 RAs. Chen et al.^112^ identified 5 randomized controlled trials in diabetic and/or Parkinson’s Disease patients (*n* = 2071) prescribed exenatide or liraglutide and reported a small but significant reduction in associated depressive symptoms in secondary analyses as compared to placebo or other antidiabetics such as insulin, sulfonylureas or pioglitazone. The presence of subthreshold depressive symptoms in the included samples may account for the small observed effect sizes (SMD = −0.12, 95% CI = −0.21 to −0.03). A separate meta-analysis, focusing on GLP-1 RAs specifically to treat major depression (6 randomized trials, *n* = 399), reported a small effect size that just fell short of statistical significance (SMD = 0.25, 95% CI = −0.1 to 0.60).^113^ Exploratory subgroup analyses in the latter study suggested ethno-geographic variability as a moderator of GLP-1 RA antidepressant response.^112^ ^,^^113^

## Concluding remarks

We propose that IRAs, via their effect on, NNA hold tremendous promise as therapeutics across multiple mental disorders. In addition, extant evidence provides a rationale for potential preventative effects, especially as it relates to cognitive dysfunction and possibly depressive symptoms with these agents. There is a need for target engagement studies in human clinical populations with these agents to better characterize the NNA effects on circuit and network connectivity. There is also the need for large, adequate, well-controlled phase II and phase III studies with these agents in the disorders that are lead candidates (eg, Parkinson’s Disease, major neurocognitive, alcohol use, and depressive disorders). In addition, preliminary evidence suggests allelic variants for gene barriers so the GLP-1 receptor gene may be associated with risk for select mental disorders (eg, alcohol use disorders, Alzheimer’s Disease).^114^ ^,^^115^

Both the United States Food and Drug Administration (FDA) and the European Medicines Agency (EMA) have issued public statements that no causal evidence exists linking IRAs to suicidality.^116^ ^–^^120^ Moreover, replicated results from cohort observational studies also suggest that GLP-1RAs are associated with no increase in suicidality and in some reports a decrease reporting in suicidality and/or conditions associated with suicide (eg, depressive disorders).^110^ ^,^^121^ ^–^^128^ In addition, there is a need for adequate well-controlled studies targeting psychotropic drug-related weight gain (PDWG) and metabolically associated comorbidity (eg, cardiovascular disease, metabolic dysfunction associated with steatotic liver disease; MASLD).^129^ ^,^^130^ The greater effect size of GLP-1/GIP co-agonists when compared to GLP-1 RAs on body weight reduction and associated metabolic morbidity, along with the direct NNA effects documented with GIP, introduce the rationale that incretin co-agonists may have additional but different mechanistic effects on systems subserving psychopathological domains.^131^
